# Supplementary material for: A SARS-CoV-2 Vaccine Designed for Manufacturability Results in Unexpected Potency and Non-Waning Humoral Response
Source: Vaccines (Basel). 2023 Apr 12;11(4):832. doi: 10.3390/vaccines11040832 (PMC10145385; doi:10.3390/vaccines11040832)
Supplement: Supplementary file 1 [file vaccines-11-00832-s001.zip › vaccines-2306584-supplementary (lack Table S1).pdf]

Supplemental Data

A SARS-CoV-2 vaccine designed for manufacturability results in unexpected potency and non-waning humoral response

Elliot Campbell[1,2,‡], Julie Dobkin[3,4,‡], Louis J. Osorio[3], Afsal Kolloli[5], Santhamani Ramasamy[5], Ranjeet Kumar[5], Derek B. Sant'Angelo[3], Selvakumar Subbian[5,*], Lisa K. Denzin[3,*], Stephen Anderson[1,2,6,*]

[1]Center for Advanced Biotechnology and Medicine, Rutgers University, Piscataway, New Jersey 08854
[2]Macrotope, Inc., Princeton, NJ 08540
[3]Child Health Institute of New Jersey, Department of Pediatrics and Pharmacology, Rutgers Robert Wood Johnson Medical School, New Brunswick, NJ 08901
[4]Graduate School of Biomedical Sciences, Rutgers, The State University of New Jersey, New Brunswick, NJ 08901
[5]Public Health Research Institute (PHRI), New Jersey Medical School, Rutgers University, Newark, NJ 07103
[6]Department of Molecular Biology and Biochemistry, Rutgers University, Piscataway, NJ 08854
‡These authors contributed equally
*Corresponding authors:
Email address: anderson@cabm.rutgers.edu (S. Anderson)
Email address: denzinlk@rutgers.edu (L. K. Denzin)
Email address: subbiase@njms.rutgers.edu (S. Subbian)

LIST OF SUPPLEMENTAL FIGURES

Figure S1. Example DisMeta Output

Figure S2. Expression and Purification of MT-001

Figure S3. Hamster challenge data

Figure S4. External validation

Figure S5. Sandwich ELISA format

(Available in a separate file)

Table S1. Structural alignment of selected members of the coronavirus superfamily

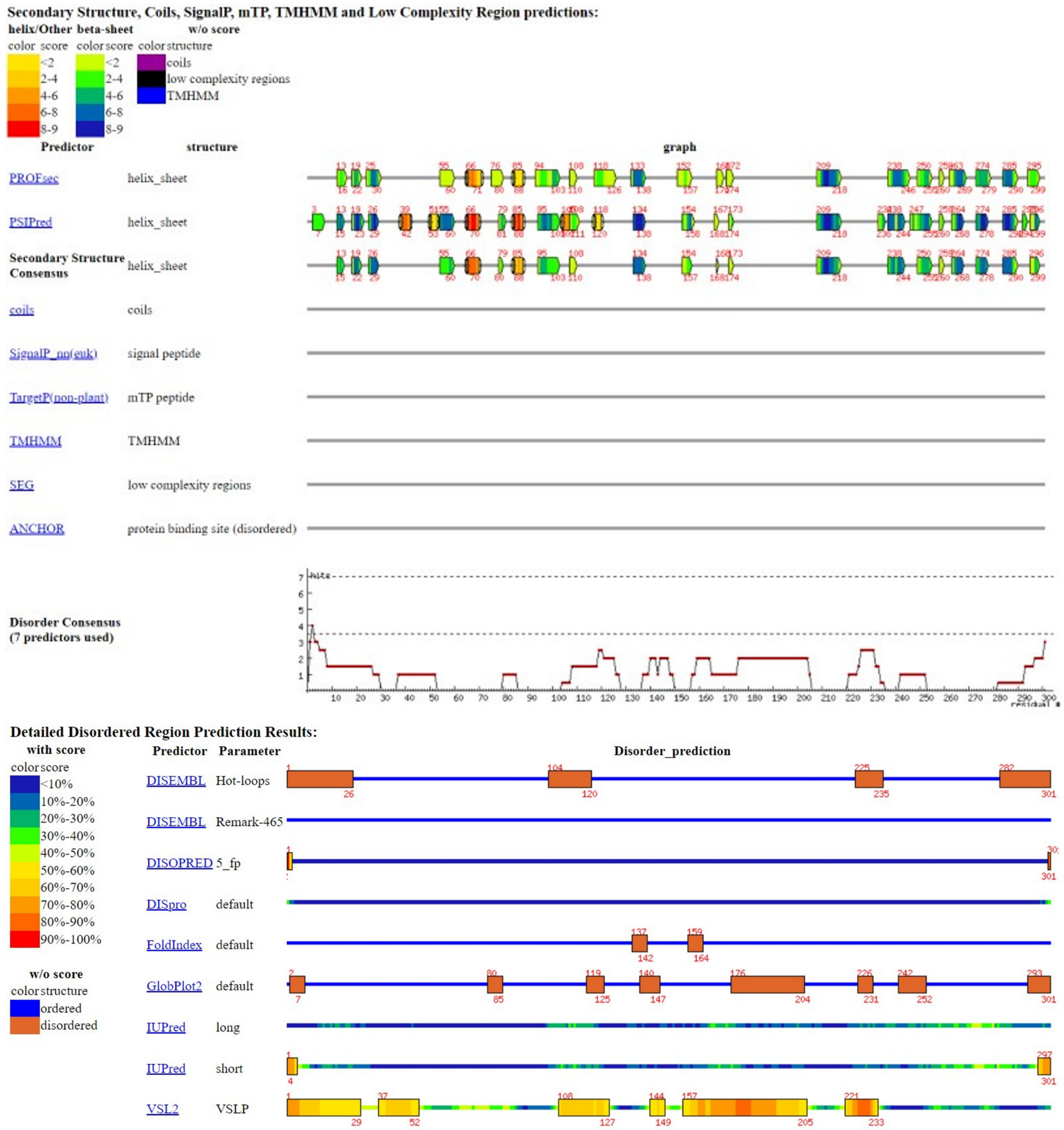

Figure S1. Example of DisMeta output for the SARS-CoV-2 spike protein region from residues 300-600 [Note: these residues correspond to numbers 1–301 in the above plots]. This tool aggregates a number of biophysical protein predictor algorithms to help visualize protein structural elements and identify domain boundaries even in the absence of detailed structural information. MT-001 comprises an ordered domain in the region from 316-594 (17-295 in the above output).

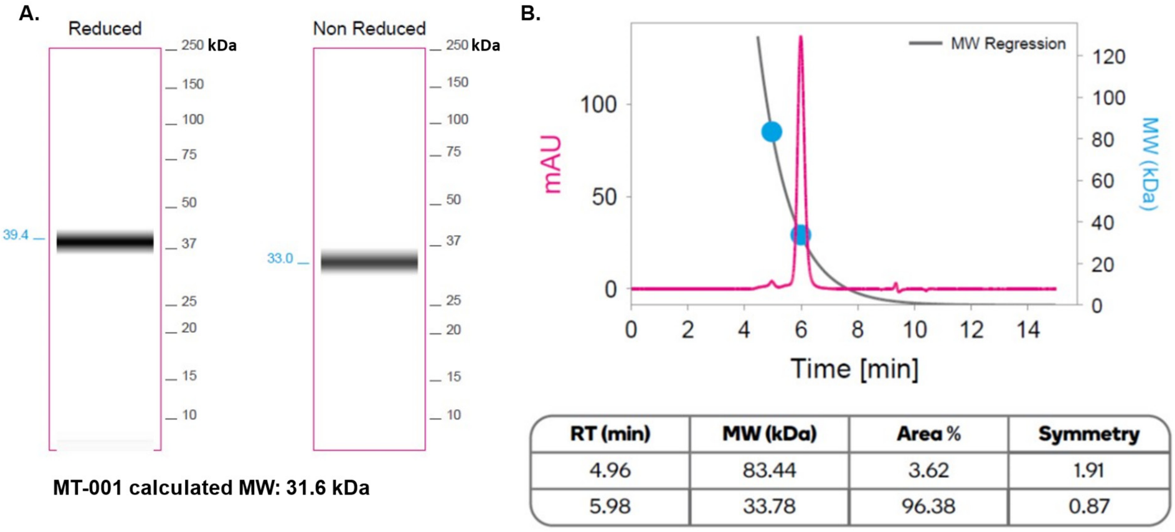

MT-001 calculated MW: 31.6 kDa

RT (min)	MW (kDa)	Area %	Symmetry
4.96	83.44	3.62	1.91
5.98	33.78	96.38	0.87

Figure S2. Expression and Purification of MT-001. MT-001 was transiently expressed in 1L HEK293 suspension culture and purified in a single affinity chromatography step using CaptureSelect C-tagXL resin. Purified MT-001 was buffer exchanged into PBS containing 10% glycerol, and protein quantity, concentration, and purity were determined by UV-VIS spectroscopy and capillary electrophoresis, and oligomeric state was determined by HPLC-SEC. The purified protein was found to be predominantly monomeric (estimated size 33.78 kDa), with an apparent molecular weight by capillary electrophoresis under reducing conditions of 39.4 kDa (calculated 31.6 kDa) consistent with a glycosylated protein. The final purified yield of MT-001 was 160.49 mg. Data was generated by ATUM, Inc.

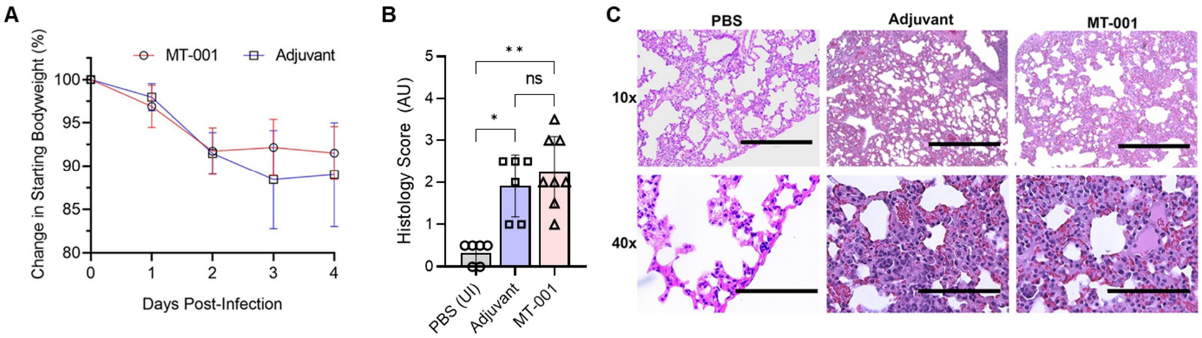

Figure S3. **Hamster challenge data.** Hamsters were immunized twice at a three-week interval with 10 µg MT-001, 500 µg Alhydrogel, and 100 µg ODN1826 or a mock-vaccination control where the MT-001 antigen was replaced by PBS. Six-weeks post-primary immunization, hamsters were challenged intranasally with 10^5 PFU of SARS-CoV-2 US/WA-1. A. Body weight was monitored daily from the time of infection (T=0) until four days post infection and represented as percentage change between T=0 and 4 days post infection.. B. Histological scoring of the MT-001 or Adjuvant vaccinated and SARS-CoV-2 infected hamster lungs at 4 days post infection. * P<0.05; **P<0.01; ns-not significant. C. Representative H&E stained lung section of MT-001 or Adjuvant or PBS vaccinated and SARS-CoV-2 infected hamster lungs at 4 days post infection at 10x or 40x magnification. Scale bar at 10x represents 400 µm and 40x represents 100 µm.

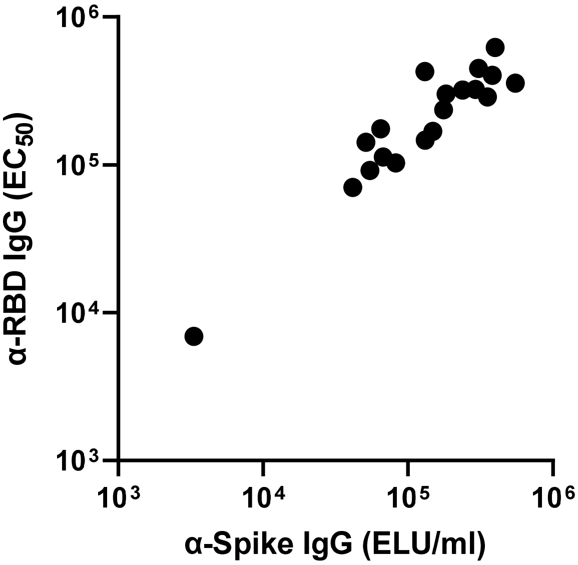

Figure S4. External validation -- Correlation with third party-generated data on the same samples. An initial experiment was performed with female BALB/cJ mice (n=5) immunized with low (1 µg) or high (15 µg) concentrations of MT-001. Varying doses of MT-001 (1 or 15 µg) were formulated with 500 µg Alhydrogel® (alum) and administered as two intramuscular (IM) injections at a 3-week interval (Figure 2A). This prime-boost regimen recapitulated the original immunization schedule utilized for human COVID-19 vaccines to promote a quick immune response [83]. To determine immunogenicity, sera was collected at 5-7 weeks following the primary immunization and MT-001-specific IgG antibody levels were assessed using a novel sandwich enzyme-linked immunosorbent assay (ELISA) that maintained 3D-conformational epitopes on RBD (See Figure S5). RBD-specific IgG half maximal geometric mean titers in the range of 1 – 3 x 10^5 were observed (Figure 2B). To determine if the MT-001 vaccine elicited a strong antibody response to RBD in the context of the entire SARS-CoV-2 spike protein and to validate our results, the same sera were also independently analyzed by the CEPI centralized laboratory network [84]. The resulting anti-spike IgG titers were similarly robust with significantly increased antibody levels (P = 0.0028) in mice immunized with 15 µg MT-001 when compared to mice immunized with 1 µg. For all mouse sera tested, the ELISA results for our in-house indirect RBD binding assay were highly correlated with the independently performed anti-spike IgG binding assays.

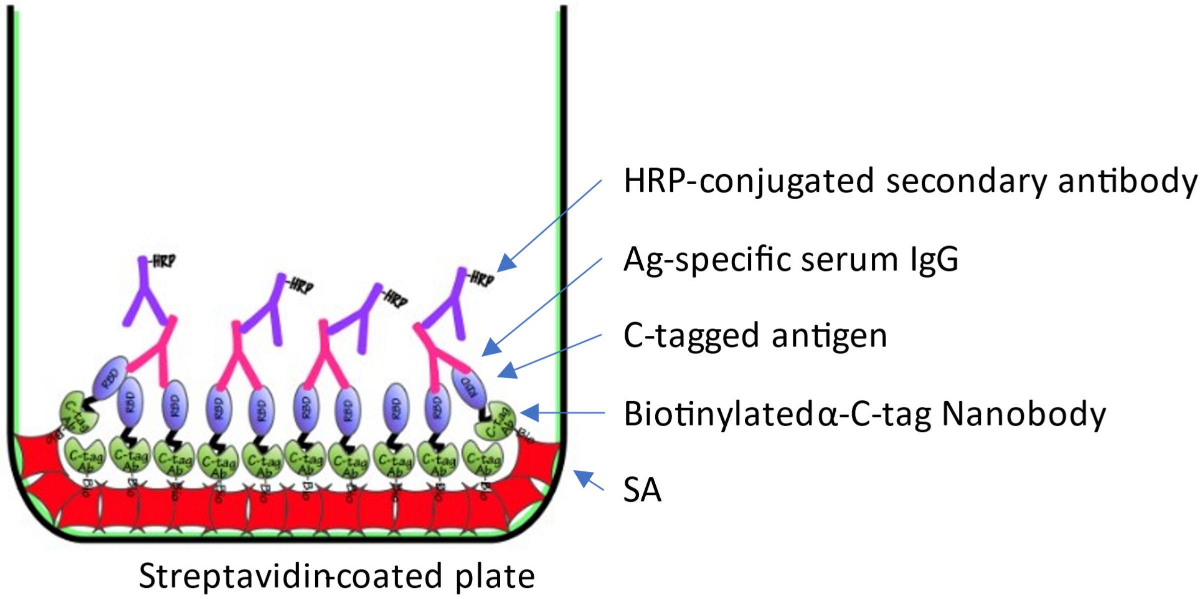

Figure S5. Sandwich ELISA displaying 3D-conformational epitopes. Anti-MT-001-specific IgG antibody levels were assessed using a sandwich enzyme-linked immunosorbent assay (ELISA) that was designed to display native 3D-conformational epitopes on RBD. Biotinylated anti-C-tag nanobody (ThermoFisher) was immobilized on streptavidin-coated microwell plates and used to capture C-tagged MT-001 antigen.

(Available in a separate file)

Table S1. Multiple sequence alignment of SARS-CoV-2 spike residues 300-600 with other members of the coronavirus family. Representative sequences were aligned using PROMALS3D (http://prodata.swmed.edu/promals3d) utilizing available structural information to ensure accurate domain alignments.
